# Recent Advances in Coarse-Grained Models for Biomolecules and Their Applications

**DOI:** 10.3390/ijms20153774

**Published:** 2019-08-01

**Authors:** Nidhi Singh, Wenjin Li

**Affiliations:** 1 Institute for Advanced Study, Shenzhen University, Shenzhen 518060, China; 2 College of Physics and Optoelectronic Engineering, Shenzhen University, Shenzhen 518060, China

**Keywords:** molecular dynamics simulation, coarse-grained modelling, multiscale simulation, biomolecules

## Abstract

Molecular dynamics simulations have emerged as a powerful tool to study biological systems at varied length and timescales. The conventional all-atom molecular dynamics simulations are being used by the wider scientific community in routine to capture the conformational dynamics and local motions. In addition, recent developments in coarse-grained models have opened the way to study the macromolecular complexes for time scales up to milliseconds. In this review, we have discussed the principle, applicability and recent development in coarse-grained models for biological systems. The potential of coarse-grained simulation has been reviewed through state-of-the-art examples of protein folding and structure prediction, self-assembly of complexes, membrane systems and carbohydrates fiber models. The multiscale simulation approaches have also been discussed in the context of their emerging role in unravelling hierarchical level information of biosystems. We conclude this review with the future scope of coarse-grained simulations as a constantly evolving tool to capture the dynamics of biosystems.

## 1. Introduction

Biological systems are now seen as molecular machines consisting of hundreds up to thousands of atoms. Molecular dynamics simulation is the biophysical microscope to study this complex molecular machinery. In the 1970s, Martin Karplus, Michael Levitt and Arieh Warshel laid down the foundation for the development of multiscale models for complex chemical systems. For their significant contribution, they were awarded the Nobel Prize in chemistry in the year 2013. Their well-recognized work included the coarse-grained modeling of proteins. They introduced the concept of simplification of biomolecular complexes for longer simulations at biological relevant time scales [1]. Based upon the concept of simpler representation, many coarse-grained models have been proposed and are being extensively used to explore biomolecular complexes [2,3,4].

**Figure 1:**
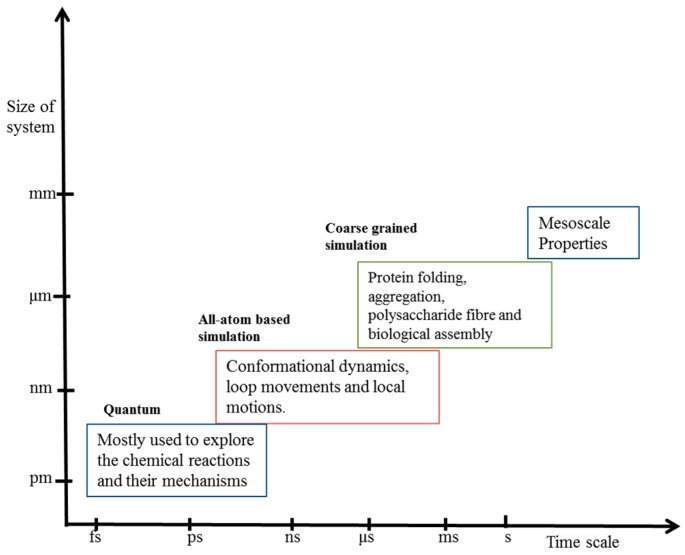
The Molecular Dynamics Simulations at different levels of resolutions (Quantum, all-atom and coarse grained) and their applicability to approximate size of biological systems and time scale.

The numerous static snapshots when put together can narrate the story very well in the form of a movie. Similar is the case for biological processes, in that when the static information of biomolecules is combined with dynamic studies it can unravel the varied aspects of complex biomolecular mechanisms. The biological system is hierarchical in nature and, therefore, multiscale approaches have been developed to address the study of biological processes [5]. The chemical reactions and their mechanisms are treated with quantum chemical calculations [6]. However, the high computational cost of quantum calculations limits its use up to fewer atoms and short time scales. In the next level, classical all-atom molecular dynamics (MD) simulations are in regular practice to gain insight into the conformational dynamicity of proteins and their interactions with nucleic acids, ligands and other proteins [7,8,9]. However, some biological processes such as protein folding, aggregation and biological assembly occur in longer time scales beyond the scope of all-atom simulations. Therefore, processes involving large-size biological systems and longer time scale dynamics are approached by coarse-grained simulations. The simulation methods used at various time and length scales are shown in Figure 1.

The all-atom simulations and coarse-grained model-based simulations are closely interwoven with each other. Thus, we will provide an overview of all-atom molecular dynamics and applications followed by a discussion on the necessity of coarse-grained methods. In the next section we will discuss the recent developments in coarse-grained methods for proteins and carbohydrates and their applications. In the third section the most recent applications of coarse-grained methods for biomolecular complexes has been reviewed while the discussion on multiscale models is given in the fourth section. In the fifth section, a brief discussion on the challenges in coarse graining of biomolecular systems is also given. Finally, a short conclusion has been made portraying coarse-grained methods as a promising one for the studies of biological systems. Due to the limited space, many important topics such as details of fundamental principles used to construct coarse-grained models, their applications to nucleic acids, water and lipids are not mentioned. For the reason not to be exhaustive we have focused on the most recent applications of coarse-grained methods published in the last five years.

### 1.1. All-Atom Molecular Dynamics Simulations

At present, the protein databank consists of more than 152,500 entries for protein and nucleic acid structures [10]. This enormous structural wealth and the study of its dynamicity can lead us to the next level of understanding of the complex biological phenomena. The availability of experimental data, enhanced computational power and improved algorithms has made the molecular dynamics simulation technique a popular choice to study the biological processes [7].

All-atom MD simulations can be done to capture the motion of atoms to study conformational changes, the process of ligand binding, protein–protein interactions, and the protein nucleic acid interaction effect of perturbations such as mutation, protonation and phosphorylation. In silico perturbations can be implied to study the biomolecular system, such as removal of bound ligand from the reported experimental structure and then study its effect on protein conformation [11,12]. All-atom MD simulations can also be used to study the post translational modification, such as the effect of phosphorylated amino acid residue [13].

Accuracy and transferability are crucial aspects for the successful application of all-atom force fields in biomolecular simulations. Although recent years have witnessed substantial improvement in the all-atom forcefields [14,15,16,17,18,19], they are still imperfect, and the resulted uncertainty should be considered carefully for the interpretation of simulation results. For in-depth discussions on their strength and drawbacks, we direct the reader to several excellent reviews [20,21,22].

Another important aspect of relevant MD simulation in studying biological processes is the length of the simulation run. The biological processes occur in the time scale of nanoseconds or microseconds (loop closing—10 ns; α-helix formation—200 ns; and mini protein folding—1 to 10 µs). Keeping in view the numerical stability of a system, the time step is usually set in range of femtoseconds during the MD run. Therefore, simulation of an inherently large biological system at a biologically relevant time scale has very high computational cost. To address this issue, nowadays graphics processing unit (GPU)-enabled simulations are being run at less cost in terms of time [23]. For instance, the DHFR system consisting of 23,558 atoms when simulated over a single NVIDIA GTX-TITAN GPU card achieved a maximum speed of 110.65 ns/day [24].

### 1.2. Enhanced Sampling Methods: An Effort to Understand the Larger Picture of Biological Processes

In recent years, the availability of experimental structures has helped MD simulation to flourish and address the protein structural and functional studies through conventional all-atom MD simulation. However, it is still a challenging task to study the thermodynamic properties of the protein-ligand system or protein folding process. To combat this issue, enhanced sampling methods have been developed to calculate the free-energy landscapes. The binding strength is quantified through the difference in energy between the free and the bound state of a system. Various methods, such as umbrella sampling, parallel tempering and multi canonical MD simulation, have been reviewed in detail by Jinzen Ikebe et al. [25]. Apart from these, Replica Exchange Molecular Dynamics (REMD) is an enhanced sampling technique, used frequently for studying the folding thermodynamics of disordered proteins [26]. The main idea of REMD simulations is to perform concurrent simulations for *n* different replicas under different condition to liberate the structure from local minima. Several parallel simulations will also allow the study of transition states to be precisely understood. Alpha-synuclein has been well studied for its association with neurodegenerative diseases. The missense mutation A53T is known for causing misfolding of the protein. Coskuner et al. [27] has studied the effect of this mutation with the help of REMD simulations. They have calculated the secondary structure conversion and Gibbs free energies for the A53T mutant and native α-synuclein. This study helped to propose the role of amino acid residues involved in the aggregation process as well as guidance for the design of specific inhibitors. Markov state models have emerged as an advanced and statistically sound method to track the entire dynamics of a system existing in thermodynamic equilibrium. The Markov state models are represented by a transition probability matrix which encloses all the metastable states in the configurational space of the system. The Markov state model methods and their applicability have been reviewed in detail by Brooke E. Husic [28].

### 1.3. Coarse-Grained Modeling and Necessity

Given the various available simulation methods and computational power in recent time one can simulate systems consisting of 10^7^ atoms in up to one microsecond [3]. The simulation of large-size systems is computationally expensive. For example, with an increase in the length of a membrane system the computational cost increases substantially due to the required computational power for the equilibration step [29]. Moreover, biological processes can occur in the range of microseconds to seconds and beyond. Therefore, in order to capture the events close to a biological relevant time scale, coarse-grained models seem to be a viable method. The study of the mesoscopic properties of a system through several microscopic events can create a lot of noisy data. Alternatively, coarse-grained models are suitable to study the mesoscopic properties of the system [30]. They offer the platform to bridge the gap between all-atom based simulation studies and the macroscopic behavior of biological mechanisms. The coarse-grained (CG) models are well suited for the study of large scale biological complexes such as ribosomes, cytoskeletal filaments and membrane protein systems. Coarse-grained models have evolved as a powerful tool for the study of biological systems for a long range of time scales up to milliseconds [31].

## 2. Coarse-Grained Simulation: Basic Principle

Coarse-grained models are a reduced representation of all-atom models that retains the essential molecular aspects for the system of interest. The reduced representation of atoms allows the simulation of large-scale biological systems. The coarse-grained models enable faster sampling due to reduced degrees of freedom. Consequently, simulations can be done at longer time scales. Coarse-grained simulations provide access to the in-depth knowledge of dynamics of macromolecules which is beyond the scope of all-atom simulations. In this section we will summarize the principles of CG simulations, the popular coarse-grained models, and the recent progress and application of coarse-grained models citing the work done for various macromolecular complexes.

In principle, development of CG models requires (a) defining of pseudoatoms sites which represent the group of multiple atoms; (b) derivation of the energy function *U_CG_* for the models which defines the interaction between pseudoatoms. It should reproduce the thermodynamic properties of the system referred; and (c) defining dynamical equations to study the time-based evolution of the CG system. The derivation of energy function to effectively define the interaction between the reduced coarse-grained sites has gained much attention and has been broadly classified into structure-based, knowledge-based and physics-based methods [4]. The structure-based approaches utilize experimental data of native conformation to define CG site, usually based upon the position of the Cα atom. The energetics of these models can be defined by network models [32] and (off-lattice) Gō models [33]. These models do not deviate much from the native state, therefore they are not able to track large conformational changes. To address this, variants of these models have been developed. For example, the self-organizing polymer model (SOP), a variant of Gō models, has been successfully applied to proteins and nucleic acids [34,35]. The knowledge-based approach includes the CG model parametrization based upon solved experimental structural data [36]. They possess a higher degree of transferability and can be applied to any system of interest. These models are primarily used for protein structure prediction [37]. In contrary to the above-mentioned approaches, physics-based methods utilize statistical data from all-atom MD simulations to derive a CG mapping scheme through systematic algorithms [38,39]. Here we present in brief about the widely used coarse-grained models with an emphasis on protein and carbohydrate models and their successful applications to biological complexes. Table 1 gives an overview of the recent progress made in the coarse-grained models and their applications.

### 2.1. Coarse-Grained Models for Proteins

Coarse-grained models have been extensively used for studying the protein folding mechanism, structure prediction, protein-membrane systems and aggregation. The coarse-grained protein model development is consistently evolving, and their applications are widely used. There is an excellent review on details of coarse-grained models of proteins by Kmiecik et al. [2]. In this section we have reviewed the most recent progress in popular coarse-grained models for proteins and their applications.

#### 2.1.1. MARTINI Model

**Figure 2:**
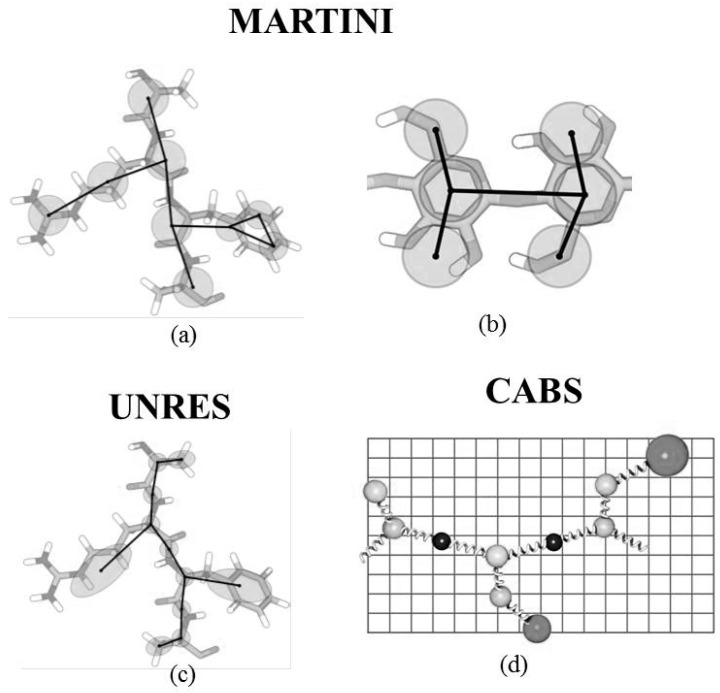
(**a**) The peptide Ala, Arg, Phe and Ala is represented as coarse-grained (CG) beads based on the MARTINI force field. (**b**) The CG mapping of the saccharide unit based upon the MARTINI force field. (**c**) The CG beads for peptide based upon the United Residue (UNRES) model. (**d**) The CG beads based upon the C-Alpha, Beta and Side Chain (CABS) model (adapted from References [2,3]).

The popular coarse-grained model for studying membrane proteins is MARTINI [71]. It was initially proposed for lipids and later extended for protein systems. The MARTINI model follows one-to-four mapping in which four heavy atoms and hydrogen associated to them corresponds to one interaction site (Figure 2a). Therefore, one coarse-grained bead for water represents four molecules of water while side chains of aromatic amino acids are represented by a higher resolution. The model is relatively simple and consists of mainly four types of interaction sites—nonpolar, polar, apolar and charged. These are further categorized into subtypes on the basis of hydrogen bonding capability and polarity. The non-bonded interaction term is calculated by the Lennard-Jones 12–6 potential and the bonded term is defined by the standard potential energy function [72]. Recent applications of MARTINI models for the study of proteins were reported which includes the protein-ligand binding process [73], mechanism of allostery [40], predicting the binding mode of peptides to G-Protein coupled receptors [41] and determining the role of hydrophilicity and hydrophobicity in the self-assembly of (AF) 6H5K15 peptide derivatives [74].

The MARTINI model has been successfully and extensively applied to study the membrane proteins and their association with lipids. For example, a coarse-grained MD simulation of membranes consisting of 64 molecules of rhodopsin was carried out by Periole et al. to the timescale of 100 μs [75]. This study resulted in the identification of favored interfaces for the interaction between dimer involving helices (H) 1/8, 4/5 and 5. In another study, Provasi et al. carried out extensive coarse-grained MD simulations for the opioid receptors subtypes δ, κ and μ, to derive the preferable supramolecular organization in a cell membrane model [76]. They have also reported the kinetics of receptor association. The simulation study done by Sharma et al. reported the structural study of the TCRα-CD3ε-CD3δ transmembrane domains and assembly in membranes as well as micelle environments [77]. Therefore, the model for activation of immune receptors in the membrane environment was proposed. The MARTINI model has also been used to study the role of cholesterol in the domain registration in plasma membrane [78]. Recently, Hirano et al. reported the MARTINI force field parameters for an ATP molecule and applied it to study ATP-induced dimerization of nucleotide binding domains of maltose transporter [42]. It extended the utility of the MARTINI model to study the ATP-driven functions of other ATPases such as ABC transporter proteins, myosin and kinesin. The coarse-grained MARTINI model with improved parameters to study the helical hinge regions in transmembrane proteins has been reported [79]. This model was used to study the deactivation of the β2 adrenergic receptor and motions related to KcsA potassium channel. Hinge related movements were observed and found to be in agreement with the experimental findings. Thus, the model can help in better understanding the functional dynamics of protein membrane systems. In the latest development, web servers to conduct the coarse-grained modeling of membrane proteins has been launched, thereby providing easy and wide access to coarse-grained simulations [43]. The MARTINI model has evolved as a simple, fast and flexible choice for studying a wide range of biomolecular interactions. Interested readers can refer to the article by Marrink and Tieleman [72]. They have discussed in detail the parameterization, applicability as well as the limitations of the MARTINI model.

#### 2.1.2. UNRES Model

United Residue (UNRES) is a physics-based model of medium resolution available as a webserver [80]. It is a highly reduced model for protein as each residue is represented by two interaction sites, one for the united side chain and the other for the united peptide group (Figure 2c). Therefore, it gives a 1000–4000-fold speed up as compared to all-atom MD simulations. The force field is based upon solid statistical mechanics and various MD-related methods have been used for sampling [39]. It had been extensively used for protein folding studies, protein structure prediction, protein-DNA interaction, signaling mechanism, action of chaperones and amyloid formation [81,82,83,84,85]. In an effort from Sieradzan et al., steered molecular dynamics was implemented to the UNRES force field [44]. It offered an edge to attain pulling speed comparable to the experiments. Therefore, protein unfolding pathways can be studied better as tested by the simulations conducted for the Fn3 domain of the contactin 1 human protein. The UNRES model has been used in protein structure prediction as evident from the participation in CASP experiments [86]. Recently, it performed well in CASP 12 tests with 80% of the predicted models being in the top 50% of submitted models. This method performed well on oligomeric targets. In addition, the reoptimized UNRES model has been used to select the best protein models amongst the pool of models [45]. The deep feed-forward neural network method was used to reoptimize by adding more descriptive features to the UNRES model. Recently, the server based upon the UNRES package has been made publicly available [46]. It allows local energy minimization and varied replica exchange dynamics. It is a user-friendly server as it takes protein sequences, restraints and other parameters from user input and results are displayed graphically. In another work by Sieradzan et al., a coarse-grained model for the protein and nucleic acid system based upon UNRES and the NARES-2P force field has been developed [87]. The reported model utilized the energy functions from UNRES and the NARES-2P model while the protein–nucleic acid interactions were defined from their previous work [39]. As an application of this model, the initial state of DNA damage repair has been studied. The dynamics of a Ku heterodimer bound to mismatched base pairs of DNA and binding of DNA to the MarA protein was studied. Notably, this model has been included in the UNRES software package.

It is well known that the UNRES force field has a key contribution in the area of protein structure prediction and protein folding studies. There are numerous studies reported based upon the UNRES model for understanding large protein motions and folding of small to medium size proteins [88,89]. In a step ahead, the revised backbone-local and correlation terms have been imparted onto the UNRES force field to further improve ab initio modeling of proteins and other molecules [90]. In the most recent development, the UNRES force field has been developed for the phosphorylated amino acid residues and was successfully tested for ab initio simulation of mini proteins containing phosphorylated residues [47]. Therefore, it extends the potential and use of the UNRES force field to the proteins involved in signaling pathways.

#### 2.1.3. CABS (C-Alpha, Beta and Side Chain)

CABS is medium resolution knowledge-based reduced representation model for proteins [91]. The amino acid residue is represented by four united groups—Cα, Cβ, the center of mass of the side group, and the center of the peptide bond. The position of Cα atoms is confined to a cubic lattice of a grid equal to 0.61 Å. This Cα traces works as variable to define the position of other interaction centers. The position of the side chain is dependent on the Cα−Cα−Cα angle of the main chain and the amino acid type (Figure 2d). The positions derived are based upon the statistical analysis of known protein structures. Various Monte Carlo schemes are used for sampling. The force field used is based upon several potentials of the mean force obtained from the calculated structural correlations of solved protein structures. It has been successfully applied for the simulation studies of protein folding [92,93,94,95].

The CABS model has been implied for protein structure prediction [96]. The CABS-flex server is available for the simulation of globular proteins [97]. Recently, it has been updated to version 2.0 and is publicly available [98,99]. The updated version included the applicability of methods to multimeric proteins and the option to customize the restraints and parameters related to simulation. The CABS model is also used as part of Aggrescan3D method-based server for predicting the aggregation propensity scale for folded globular proteins under the dynamic mode calculations [100]. CABS-flex methodology has been adapted to model the protein–peptide interaction for docking simulations [48]. In a similar work, the CABS model has been used to trace the large-scale changes associated with p53-MDM2 interaction [101]. In the latest development, a Python-based CABS-flex standalone package has been released. It can be used for the study of dynamics of disordered proteins, structural flexibility and folding mechanism of proteins [49].

#### 2.1.4. PRIMO (Protein Intermediate Model)

The PRIMO model is of higher resolution and developed for proteins and nucleic acids. This model includes three to eight interaction sites representing one amino acid residue and twelve to thirteen interaction sites for representation of one nucleotide. The interaction site scheme is close to atomistic representation. The energy function includes terms similar to a standard MD force field. In addition, it consists of bonded terms to define bond geometry, potential for explicit hydrogen bonding and a generalized born parameter-based implicit solvent model. PRIMO can attain about an 8 to 12 time speedup as compared to all-atom MD simulations. The model has been tested for the study of ab initio folding of small peptides and the prediction of protein structure [102,103]. The PRIMO model has an edge in its design regarding the transferability to other systems. For instance, PRIMO-M has been implied to study the membrane-bound peptides [50].

#### 2.1.5. OPEP (Optimized Potential for Efficient Structure Prediction)

This model was developed by Derreumaux and coworkers based upon knowledge- and physics-based mixed kind of potential [104]. It is a model with representation of backbone as all-atom and side chains as a single bead with an exception of proline represented by three beads. The development and rise of the OPEP model have been reviewed earlier by Fabio Sterpone et al. [105]. This model has been used for protein folding, aggregation studies, role of hydrodynamics in peptide aggregation and modeling of protein structure. Here we have discussed the OPEP model in view of recently reported studies. In a study for the Aβ16−22 peptide, it was defined by a flexible OPEP model and the initial aggregation phase study was done using multi-scale Lattice Boltzmann molecular dynamics. The hydrodynamic interaction effect was studied and found to enhance the aggregation process [106]. In another work, the OPEP model has been implied to study the amyloid fibril formation along with interactive simulations for peptide folding and response to mechanical stress caused by the surrounding fluid by catch bond proteins [105]. In a similar subject of an amylin oligomer study, the OPEP model was coupled with replica exchange molecular dynamics to study the mutation effect on its structural and thermodynamic properties [51]. The applicability of energy functions derived from the OPEP model has been extended to the prediction of score for protein–protein complexes [52].

The studies discussed above reflects the successful application of the OPEP model for studying the self-assembly of amyloid proteins, the structure prediction of peptides and the effect of hydrodynamics on protein dynamics. However, the disadvantage associated with it is that the integration step can use a time step of 2 fs, which is much lower than other coarse-grained models. Additionally, there is also the scope of further improvement in studying systems at varied pH conditions. OPEP version 6 has been recently reported by Barroso da Silva et al. [53]. The developed model was tested for the structural properties of insulin at varied pH conditions and was found to be in agreement with the experimental data [53].

#### 2.1.6. PRIME

PRIME is a medium-resolution protein model developed by Cheon and coworkers [107]. In this model, amino acid residue is represented by three spheres for backbone including NH, CαH and CO, while the side chains are divided into 14 groups based upon the charge, size, polarity, hydrophobicity and hydrogen bond formation potential. The solvent is treated in an implicit manner. The model uses discontinuous potentials to define bonding and angular restrictions thereby enhancing the speed of simulations. The hydrogen bonds are defined by directional square-well potential, therefore, favoring the formation of α-helices and β-sheets. The model is well-suited for the study of large proteins for long time scales. The model was used to study the tau fragment fibrils [108] and Aβ16-22 peptides [109]. The model was able to show conversion of oligomers of Aβ17–42 peptides to protofilaments [110]. Notably, this model has also been successful in describing the effect of crowding on amyloid beta (16-22) aggregation [111,112] and the inhibitory effect of polyphenols on Aβ fibril formation [54]. Recent applications of this model include the study of structural and kinetic details of co-aggregation of Aβ40 and Aβ16–22 peptides [113] and cross seeding in fibrillation of prion protein peptides [55,114]. These findings can be helpful in better understanding the molecular assembly of peptides and further for therapeutic purposes.

#### 2.1.7. Bereau and Deserno Model

The coarse-grained model for protein was developed by Bereau and Deserno [115]. The amino acid residues are represented by four beads. The backbone is represented by three beads, N, Cα and C′, while the side chain is represented by single bead at Cβ. The solvent is treated implicitly. The geometric parameters were borrowed from previous peptide models [116,117,118]. The bonded interactions were defined by harmonic potential. The non-bonded interactions for the side chain were defined using potential developed by Miyazawa and Jernigan [119]. The hydrogen bonds were modelled implicitly, and the interaction potential was taken from Irbäck et al. [118] based upon the 12-10 Lennard-Jones potential along with the angular term. The model also contained an extra term for dipole–dipole interactions of neighboring residues. The model has been implemented in the ESPResSO package [120]. Applications of this model so far have been focused on the studies of protein oligomerization [121] and the unfolding process of alanine rich polypeptides [56]. In an interesting study by Bereau, the two models were cross parameterized to reproduce the various peptide–membrane phenomena [57].

#### 2.1.8. Kim and Hummer Model

The coarse-grained model for large multi-protein complexes was developed by Kim and Hummer [122]. The model was simple where amino acid residues were represented by a single bead centered at the associated cα atom. The interaction potential between the amino acid residues was calculated using Lennard-Jones type potentials and electrostatic interactions for short range and long range, respectively. This model is equipped with transferable potential energy function and has been successfully applied for simulating conformational ensembles of large multi protein systems of the ESCRT-I membrane protein complex [123,124,125], the multi domain kinase Protein C βII for structure refinement [126] and protein phosphatases [127,128]. In recent works, the Kim and Hummer (KH) model has been implied in the study of multi domain cellulosomes [58,59]. The cellulosomes are comprised of multi-domains connected with each other through linkers which are intrinsically disordered regions. The linker peptides have been simulated through the KH coarse grain model to study the effect of length of the linkers over the cellulosomes. Notably, the KH-model-based simulations have been coupled with SAXS analysis to study the highly flexible protein complexes containing lipid kinases [60,61] and membrane segments of cell adhesion proteins involved in immunological responses [129]. The KH model has been implemented in software packages such as CHARMM [130] and other simulation software for coarse-grained models [131]. It is noteworthy that Dignon and workers have utilized the KH model in LAMMPS and HOOMD software packages to study the phase behavior of intrinsically disordered proteins FUS and LAF-1 [62].

### 2.2. Coarse-Grained Models for Carbohydrates

Carbohydrates are key players involved in many biological processes including the development of diseases. Carbohydrates by virtue of their nature possess a high degree of polymerization therefore opening up the possibility of an unlimited number of sequences, linkage and degree of branching [132]. Owing to the high structural diversity, the conformational degrees of freedom for carbohydrates is high [133,134]. Consequently, development of coarse-grained models for carbohydrates possesses challenges. Here, we will discuss the popular models and recently reported coarse-grained models of carbohydrates and their role in the study of structure and dynamic aspects of polysaccharides.

#### 2.2.1. M3B Model

In an early effort by Molinero and Goddard, coarse-grained model for malto-oligosachharide in an aqueous solution was developed and termed as M3B [63]. The hexapyranose ring was represented as three beads corresponding to C1, C4 and C6 atoms of an atomistic model while the water molecule was represented as a single particle. The long-range forces were defined by a 2-body Morse function. The developed model allowed the integration time step of 10 fs, thus enabling simulations for a glucan system in microseconds on single workstations. This model turned to be helpful in studying the water–glucan systems. In another study, a similar coarse-grained model for α-D-glucopyranose was developed by Liu et al. In this model each glucopyranose ring was represented by three beads. The bonded interactions were calculated through Boltzman analysis while the force-matching approach was used for calculating non-bonded interactions [64]. This model appeared to be easy to apply to polysaccharide systems but so far it has been applied to amylose and glucose systems.

In another model proposed by Bellesia et al., the interconversion of cellulose Iβ and cellulose III_I_ was studied. Their model consisted of two energy terms: Lennard Jones and dihedral constraints affecting inter-sheet equilibrium distance and rotational states of the hydroxy methyl group. The model was successful to reproduce the structure of cellulose along with thermo mechanical feature of cellulose [135]. Next, we will discuss the most popular force field, MARTINI, which was extended to carbohydrates.

#### 2.2.2. MARTINI Model

MARTINI has been a popular choice for a CG model for proteins and it was extended to carbohydrates as reported by the work of López et al. [136]. The monomeric saccharide unit was represented using three beads (Figure 2b) and parametrization followed the same principle of reproduction of partitioning free energies of molecules for polar and non-polar phases. The bonded parameters were optimized according to the most frequent rotameric state of the glycosidic bond. The model was applied to amylose and curdlan which resulted in the reproduction of structural properties. The cryo- and anhydro-protective effect was also studied for glucose and trehalose with a lipid bilayer system, which also resulted in a correlation with the experimental and calculated properties. In another recent work by César A. López, a MARTINI force-field-based, coarse-grained model has been used to study the mechanical and physicochemical properties of cellulose Iβ, such as bending resistance of cellulose nanofibers [65]. The slightly modified MARTINI model has been used to study the Ganglioside-protein and lipid interactions [137]. The mapping from an atomistic to a coarse-grained model was retained while minor modifications were done for the bonded and non-bonded parameters to reduce the self-interactions. The model reproduced the ganglioside clustering manner as evident from atomistic level simulation. The MARTINI force field has also found application in studying the longitudinal dimension of chitin fiber, which is more than hundreds of nanometers in length [138]. The chitin monomer was mapped to three beads. The starting parameters for bonded and non-bonded terms were derived from the atomistic trajectory using Boltzmann inversion calculation. The parameters were calibrated in accordance to the structure and elastic modulus of chitin. The proposed model reproduced the crystalline α-chitin structure and opened up the possibility of studying chitin and protein interactions. In another work by Steven et al., the coarse-grained model for chitosan has been developed to study the effect of the degree of acetylation on its self-assembly in solution [66]. For chitosan polysaccharide, another CG model was developed on the basis of a free-energy landscape for glycosidic bonds [67]. The model was used to study the equilibrium properties of chitosan in solution regarding the degree of deacetylation and polymerization, ionic strength and pH. Furthermore, the MARTINI force field has also been applied to study the second virial coefficient of osmotic pressure, a thermodynamic solution attribute, for polysaccharides [139]. This work has also proposed the effect and scaling of a Lennard-Jones interaction between saccharides molecules.

#### 2.2.3. Other Models

Contrary to above mentioned multi-bead per monomer model, Srinivas proposed one bead per monomer CG model to study the conformations of Iβ cellulose [68]. The centre of mass for monomer unit glucose was mapped from all-atom simulation trajectories. The bonded interactions were taken from the ensemble of conformations while the non-bonded parameters were optimized through the Boltzmann Inversion method. In this study, the solvent was treated in an implicit fashion. Later on, their group reported a CG model for natural cellulose in an explicit water system [69]. It was observed that cellulose–water interactions have an important role in the transition of crystalline to the amorphous form of cellulose.

In another effort by Glass, a residue scale CG model was developed termed as REACH (Realistic Extension Algorithm via Covariance Hessian) [70]. Each monomer was represented by a bead and the associated force field was developed through the atomistic simulation trajectory of cellulose fibril in aqueous solution at different temperatures. This model was developed for Iβ cellulose and applied for the characterization of its elastic attributes as well as degradation as a function of length and temperature.

In a work by Poma et al., a unified CG model for polysaccharide and protein systems was developed [140]. The glucose unit was represented by one atom analogous to the representation of each amino acid residue by one bead at the Cα atom. The parameters for the CG model were derived using Boltzmann Inversion and energy-based methods which were found to yield consistent results. The non-bonded interactions were calculated as Lennard-Jones potentials. The contacts between hexaose and the Man5B protein were characterized and an effective binding energy was determined using the energy-based method. The parameters are provided in detail in the methods section of the paper. This study was found to be in agreement with the fact that enzymatic activity of Man5B reduces as cellohexaose binds to it.

In the case of carbohydrates, the coarse-grained scaling is customized in accordance to the nature of the concerned question. The coarse-grained multi bead model has been found suitable for the study of varied conformational states of polysaccharides and other chemical specificity issues as indicated by the reported works. On the other hand, the coarse-grained models representing a glycosyl residue as a single bead are more useful for the structural and dynamics studies of polysaccharide assembly. The unified coarse-grained model for a protein–polysaccharide system can be helpful in studying the process of enzymatic hydrolysis of polysaccharides which has important implications in the biofuel industry.

## 3. Coarse-Grained Models and Biomolecular Complexes

The CG models have enabled us to study the assembly of biomolecular complexes as well as the inside details of protein folding and aggregation. Dynamic assembly and disassembly are key features of biological complexes. For example, the capsid protein assembly to form a capsid shell is required for viral infectivity in case of HIV. Grime et al. have performed a coarse-grained simulation to study capsid assembly in an HIV-1 system [141]. They have studied the basic principles of capsid assembly and the effect of varied conditions such as molecular crowding, concentration of capsid assembly and the conformational changes associated with it. In another study by Pak, a coarse-grained simulation of sub-nanometer resolution has been applied to study the interaction network of HIV-1 assembly and budding of Gag polyprotein [142]. They have suggested the putative role of the Gag protein, RNA and cell membranes in the initial stages of immature HIV-1 lattice assembly. Coarse-grained simulations have also been extensively carried out to study the capsid assembly in case of HIV as evident by several reported studies [143,144,145,146,147].

Action mechanisms of motor proteins have also been studied by coarse-grained models. For instance, a coarse-grained model-based simulation study has been used to elucidate the molecular basis of motility of myosin VI in detail [148]. In this CG model, amino acid residue was represented by one bead with Cα positioned as its center. The interaction between the beads was defined on the basis of the distance between beads in the native state. This work helped to explain the motility of myosin VI in two steps: The change of the motor domain conformation, pushing the lever arm in the forward direction, and then the lever arm undergoes rotational diffusion. The simulation results were found to be in agreement with polTIRF experiments and, therefore, established the reliability and utilization of CG-based simulations. In another study, the molecular mechanism of kinesin has been elucidated [149]. The motion of kinesin on microtubules precedes processively, spanning 16 nm in each step. The MT-kin complex was defined as a coarse-grained model using the self-organized polymer model. In this the model, amino acid residue is represented by the interaction center at the cα position. Brownian dynamics-based simulations were done to determine the mechanism of the kinesin step in detail.

Coarse-grained methods are well suited for the studies of the folding process of intrinsically disordered proteins [150,151]. Recently, Ramis et al. have performed coarse-grained simulation using the SIRAH force field for the intrinsically disordered protein α-synuclein [152]. They have successfully reproduced the properties of α-synuclein and therefore contributed to the better understanding of its role in disease. CG models have also found applicability in the understanding of nanomechanical characterization of biological fibrils. The mechanical and thermodynamic properties of intrinsically disordered proteins, Aβ40, Aβ42 and α-synuclein have been investigated and found to complement the experiments [153]. To this end, CG models have also been used to study the mechanism of fibrillar growth and attachment of free monomeric units to it in α-synuclein [154]. Coarse-grained models are also being used in combination with all-atom MD. Therefore, the integrated models provide insight into the hierarchical level of information. In the next section we will discuss the multiscale MD simulations and their applications.

## 4. Multiscale Simulations and Coarse-Grained Models

The structural dynamics of biological macromolecules and underlying mechanisms spans from atomic to molecular level. In order to gain an in-depth understanding of the mechanisms, large scale motions of macromolecular complexes with an insight of atomic picture is required. Therefore, multiscale simulations have emerged as a promising approach. The coarse-grained models are integrated with all-atom models and used to define different components of a system. The study of rotary motor proteins through free-energy landscapes and CG-based simulation is a well-studied example of multiscale simulations. Toru Ekimoto has reviewed the multiscale dynamics study of rotary ATPases [5]. The human Frizzled and Taste2 GPCRs are another class of proteins studied extensively with varied simulation approaches. Multiscale simulations of this class have suggested the activation mechanism of FZD4 class receptors as well as the recognition mechanism of Taste 2 GPCRs and the effect of drugs. The various reported simulation studies have been reviewed by Prieto et al. [155]. The multi-resolution approach can help to overcome the inaccurate atomistic details of low-resolution complexes and enables faster sampling of the system. For example, Tarenzi et al. proposed the integrated MM/CG approach with an H-AdResS scheme for the solvent [156]. In this work, binding pocket and neighboring residues were treated as the molecular mechanics (MM) MM region while the rest of the protein was coarse-grained modelled. In this model, each amino acid residue was represented by single bead centered at Cα and interactions between beads were calculated through Gō-type potential. The solvent was represented through the Hamiltonian formulation H-AdResS, allowing the free diffusion of water molecules between MM and the coarse-grained region. Thus, this multi scale approach is well suited for better understanding protein ligand interactions and consequently drug design applications.

In an alternative approach, reverse mapping methods have been developed to reconstruct all-atom structures from the coarse-grained models [157,158]. The coarse-grained models can be integrated with a mesoscale-level representation of a solvent through the Lattice Boltzmann (LB) method. It is based upon the kinetic description of the solvent explained by the fluid dynamics under varied conditions. LB-based hydrodynamics in combination with the OPEP coarse-grained model for proteins have been reported to study the protein relaxation and aggregation processes [106,159,160].

## 5. Challenges in Coarse-Grained Modeling

Similar to all-atom force fields, coarse-grained models bear their own limitations. Firstly, they are simplified representations of systems under investigation by eliminating atomic details, which may exert significant effects on accurate predictions of important properties of the studied systems. Apparently, such simplified representations provide limited chemical resolution compared to atomistic models; as a quick fix, the atomic details could be reconstructed from coarse-grained models, which however is not trivial [161]. Generally, the dynamics of the system is not uniformly scaled up, which makes it difficult to evaluate the time scale of the simulated processes [162]. The coarse-grained models may have difficulties to predict the correct kinetics and thermodynamic properties [163,164].

Since coarse-grained models are tailored for specific features of systems or phenomena of interest, they have a lesser degree of transferability with respect to varied systems; that is to say, they should be applied to study specific systems or systems under proper thermodynamic conditions. For instance, the MARTINI model is based upon the calibration of non-bonded interactions against the oil/water partitioning coefficients [72]. Membrane–peptide binding and protein–protein recognition are highly correlated to this partition coefficient. Consequently, the MARTINI model has found dominant applications in studying the membrane protein systems and their interaction with lipids. PRIME is a knowledge-based coarse-grained model specially designed for discontinuous molecular dynamics simulation studies of the protein aggregation process [107]. The CABS model is exclusively designed for structure prediction or refinement [91]. Also, pH has an important role in the cell environment, especially for the enzymes. The development of the OPEP6 model has offered the study of pH-mediated biological processes. The applicable processes or questions for the various coarse-grained models have been summarized in Table 1.

The challenges in accuracy and transferability are just examples among many other challenges, such as model assessment, the integration of multiple coarse-grained models and adaptive resolution. We direct the readers to recent reviews for further discussions on the challenges of coarse-grained models [72,165,166].

## 6. Conclusions

Computational chemistry has witnessed rapid advancement in modeling the biomolecular complexes and their interactions. Molecular dynamics simulation has emerged as promising approach to dissect the biological phenomenon spanning from the atomistic level of motion to biological assemblies. Interestingly the continuing improvement in computational power and algorithmic accuracy has provided accessibility of simulations to the wider scientific community. The coarse-graining models and their applications have been discussed in this review and seem to be a suitable choice for studying biological phenomenon at a relevant time scale. The coarse-grained modeling lacks general applicability as coarse-grained methods require careful choice regarding the coarseness and energy functions as well as the sampling scheme and analysis according to the query being addressed. The lesser the number of pseudoatoms represented, the speed of simulations would be higher but the accuracy lower. The popular models using three to four beads per residue for proteins can accelerate simulation in 3–4 orders of magnitude as compared to all-atom models [165]. We also anticipate that the advent of new coarse-grained models in terms of better transferability will help to extend its applicability to the study of new phenomenon quickly and easily. After more than a decade-long history of coarse-grained modeling, it has found successful stories in the field of protein structure prediction, protein folding, studying biological assemblies, structural and dynamic aspects of polysaccharide assembly, to list only a few. Multiscale modeling of biological complexes at longer timescales can address a wide range of questions of biology [167]. Moreover, the realistic cellular environment is a mixture of various biomolecules interacting across a broad range of time and spatial scales. Coarse-grained modeling can be seen as an inclusive method for the simulation of whole-cell models [168].

## Figures and Tables

**Table 1:** Overview of recent progress in coarse-grained models and their applications.

Coarse-Grained Models	Granularity of the Model	Recent Advances, Example Application and Additional Information
**Proteins**		
MARTINI	Up to five beads per amino acid residue	mechanism of allostery [40], peptide binding to GPCRs [41], Parameters developed for ATP molecule [42] MERMAID (webserver for simulation of membrane proteins (http://molsim.sci.univr.it/mangesh/index.php) [43]
UNRES	Two beads per residue	Steered molecular dynamics integrated to UNRES [44], deep feed-forward neural network-based re-optimization of UNRES for ranking of protein structure models [45], freely accessible server launched (http://unres-server.chem.ug.edu.pl.) [46], parameters developed for phosphorylated residues [47]
CABS	Four beads per residue	CABS-dock (webserver for flexible docking of peptides) [48] CABS-flex standalone package [49],
PRIMO	Three to eight beads per residue	Provides high resolution and transferability [50]
OPEP	Up to six beads per residue	Replica Exchange MD and OPEP [51], protein–protein docking [52], OPEP 6 (Constant-pH Molecular Dynamics Simulation Scheme) [53];
PRIME	Four beads per residue	Effect of inhibitors on Aβ fibril formation [54]; co-aggregation of Aβ40 and Aβ16–22 peptides cross seeding in fibrillation of prion protein peptides [55]
Bereau and Deserno	Four beads per residue	Refined model for thermodynamics of unfolding process of peptides [56] cross parameterization of two models to study peptide-membrane [57]
Kim and Hummer	Single bead centered at cα atom.	Study of multi-domain cellulosomes [58,59]; coupled with SAXS to study highly flexible protein complexes [60,61]; phase behavior of intrinsically disordered proteins [62].
**Carbohydrates**		
M3B	Three beads per monosaccharide	Pioneer model for CG methods of carbohydrates [63]
Bellesia model	Five beads per monosaccharide	Developed to study the structural transition from cellulose Iβ to cellulose III(I) [64]
MARTINI	Three beads per monosaccharide	physicochemical properties of cellulose Iβ [65]; Chitosan and solution behavior [66]; self-assembly of polysaccharide [67]
Srinivas model	Single bead per monosaccharide	Solvent free coarse-grained model [68]; study of cellulose fibrils [69]
REACH	Single bead per monosaccharide	Developed to study the elastic properties of the cellulose fibril [70]
